# Internship engagement as a predictive pathway between an academic self-concept proxy and employability outcomes: evidence from a public undergraduate employability dataset

**DOI:** 10.3389/fpsyg.2026.1901668

**Published:** 2026-09-16

**Authors:** Xiaoyu Zhong, Kai An, Xin Zhao

**Affiliations:** 1 School of Education, Shanghai Normal University, Shanghai, China; 2 School of Education, Hainan Normal University, Haikou, China

**Keywords:** academic self-concept proxy, attention mechanism, Behavioral Internship Engagement Proxy, deep learning, employability outcomes, HAMEN model, predictive decomposition, undergraduate employability

## Abstract

**Background:**

Employability competencies in higher education reflect both academic indicators and experiential learning. This study examines whether a behavioral internship-engagement proxy provides a predictive indirect pathway between an academic self-concept proxy and employability outcomes using the Hierarchical Attention Mediated Employability Network (HAMEN). Because the source data are cross-sectional and observational, mediation is interpreted strictly as an associational predictive decomposition rather than a causal mechanism.

**Methods:**

A secondary analysis was conducted on a publicly available Kaggle dataset containing 1,200 undergraduate records of unspecified institutional and national origin. Academic Self-Concept Proxy (ASC-P) was constructed from grade point average, core-subject score, and academic consistency, whereas Behavioral Internship Engagement Proxy (IE-P) was constructed from internship and learning-exposure indicators. Data were divided into training, validation, and test sets (70%/15%/15%); preprocessing was fitted only on the training set, and HAMEN hyperparameters were selected using five-fold cross-validation. Performance was assessed using R^2^, RMSE, MAE, bootstrap confidence intervals, and prespecified hypothesis tests.

**Results:**

HAMEN achieved *R*^2^ = 0.89, RMSE = 3.84, and MAE = 2.97, exceeding Linear Regression (*R*^2^ = 0.71) and Structural Equation Modeling (*R*^2^ = 0.78). The model-based indirect predictive component associated with ASC-P through IE-P was 0.61 (68% of the total component of 0.90; 95% CI [0.55, 0.67]; Holm-adjusted *p* < 0.001), whereas the direct predictive component was 0.29 (32%; Holm-adjusted *p* < 0.001).

**Conclusion:**

HAMEN improved employability-score prediction and assigned the largest engagement attention weights to internship duration and project experience. The results indicate predictive associations only; they do not establish that academic self-beliefs or internship engagement causally produce employability outcomes because the constructs are proxy measures and the dataset provides no temporal ordering.

## 1 Introduction

Internships are integral components of higher education, particularly in applied programs that connect theoretical learning with workplace practice (Hilmi and Lukmantoro, 2020). Universities increasingly incorporate internships into curricula to strengthen employability preparation (Gillespie et al., 2020). Such experiences expose students to workplace expectations and can support professional learning (Hu, 2019). Nevertheless, internship outcomes vary substantially across individuals, indicating that academic, behavioral, and contextual characteristics are relevant to prediction (Zuo et al., 2020).

Academic self-concept is a psychological construct describing students’ subjective beliefs about their academic competence (David et al., 2020). Valid measurement normally requires self-report items designed to capture perceived capability rather than objective attainment alone (Carter, 2019). Higher academic self-concept has been associated with motivation, persistence, and learning behavior (Aun and Chee, 2020). However, grade point average and course marks primarily reflect achievement, effort, and educational opportunity; they are not interchangeable with self-belief.

Internship engagement similarly refers to behavioral, emotional, and cognitive involvement in internship learning (Thompson et al., 2020). It includes active task participation, sustained effort, reflective learning, and psychological investment (Cai et al., 2022; Zhong et al., 2020). Greater engagement is commonly associated with skill acquisition, workplace adaptation, and professional identity development (Ramaprasad et al., 2022). Objective indicators such as internship duration or project counts capture exposure and participation but do not fully measure the psychological experience of engagement.

Prior research has frequently examined engagement as a statistical pathway linking academic beliefs with internship-related outcomes (Lin and Chen, 2022). Students with stronger academic beliefs may also report greater participation in internship activities and more favorable career outcomes (Wang and Hsieh, 2022). Nevertheless, a causal mediation claim requires temporal ordering, control of confounding, and valid measurement of the psychological constructs (Asmawi, 2022). Predictive pathway analysis can identify structured associations, but it cannot by itself establish that one construct causes another (Hsu, 2022).

Internship outcomes may include skill development, employability preparedness, professional conduct, and satisfaction (Mansoor et al., 2022). These outcomes are central to applied higher education across multiple institutional and national settings (Hsu and Liao, 2022). Their variability motivates examination of academic indicators, experiential exposure, and engagement-related behavior without assuming that a public dataset represents a specific university or country (Hsu, 2022).

Accordingly, this study evaluates whether a behavioral internship-engagement proxy carries a predictive indirect association between an academic self-concept proxy and employability competitiveness in a public undergraduate dataset of unspecified origin (Hayat et al., 2020). The analysis is intended to clarify the predictive contribution of academic and experiential indicators and to assess the performance and interpretability of HAMEN (Guo et al., 2022). No claim is made that the data constitute a case study of a Chinese applied undergraduate university.

Within educational psychology, the study makes a deliberately bounded methodological and interpretive contribution: it demonstrates how objective academic-attainment and experiential-participation indicators can be used as transparently labeled proxies in a leakage-controlled predictive framework while preserving the distinction between prediction and psychological measurement. Objective indicators such as GPA, course scores, internship duration, and activity counts are not treated as substitutes for validated measures of academic self-concept or engagement; rather, the proxy terminology clarifies what secondary observational data can and cannot support in educational psychology research on employability.

The study therefore pursues six connected objectives: to quantify the association between Academic Self-Concept Proxy (ASC-P) and Behavioral Internship Engagement Proxy (IE-P) among undergraduate records; to examine the predictive association between IE-P and Employability Competitiveness Score (ECS); to estimate the direct predictive component linking ASC-P with ECS; to estimate the indirect predictive component associated with ASC-P through IE-P without interpreting it as causal mediation; to develop a HAMEN-based deep learning model for predicting employability competitiveness; and to validate model performance, robustness, hypothesis tests, and feature-ranking stability using held-out evaluation and bootstrapping.

These objectives are addressed through five core behavioral and predictive research questions: (RQ1) Is ASC-P positively associated with IE-P among the undergraduate records? (RQ2) What is the predictive association between IE-P and ECS? (RQ3) Does ASC-P retain a statistically significant direct predictive component for ECS? (RQ4) Is the model-based indirect predictive component through IE-P statistically significant? and (RQ5) Does HAMEN predict ECS more accurately than Linear Regression and Structural Equation Modeling? A sixth question, RQ6 (Which engagement indicators receive the largest and most stable attention weights?), is explicitly designated as a model-diagnostic question because it examines HAMEN’s internal attention allocation rather than a core behavioral relationship or psychological construct.

### 1.1 Study objectives and hypotheses

*H1*: ASC-P is positively associated with IE-P.

*H2*: IE-P is positively associated with ECS.

*H3*: ASC-P has a significant positive direct predictive component for ECS.

*H4*: The indirect predictive component associated with ASC-P through IE-P is statistically significant.

*H5*: HAMEN significantly outperforms the prespecified baseline models in predicting ECS, as indicated by higher R^2^ and lower RMSE and MAE.

*H6*: Internship duration and project experience receive the two largest and statistically stable engagement attention weights.

Relevant literature on internship engagement and employability is summarized below.

The research conducted by Huang et al. (2024) indicated that the internship environment in clinical settings impacts caring behavior through moral sensitivity and self-efficacy, promoting ethical and professional development. Dioquino et al. (2024) have explored such issues as internship experiences, self-efficacy, and career goals of nursing graduates; however, while self-efficacy was confirmed as an important variable, internship experience does not affect it. Kakkar et al. (2024) analyzed the issue of remote internships and established that communication at the institution reduces cynical views while faculty assistance decreases cynicism only. Tekbıyık (2024) determined that talent management processes have a positive effect on interns’ satisfaction and organizational attractiveness, which act as mediators of conversion intentions. According to Wang and Zhang (2024), self-efficacy is a factor that influences learning engagement through psychological resilience and professional commitment, with gender playing a moderating role.

Tseng et al. (2024) have shown that patient engagement plays an important role in mediating effects of discrimination and denial of health-related issues on substance abuse. In their study, Ni et al. (2024) showed that engagement of employees in their jobs helps mediate effects of job demands and performance on job efficiency. Lam et al. (2024) stated that job characteristics of internship positively contribute to job satisfaction and involvement that help shape career intentions and performance of students in the field of hospitality. The study conducted by Jin et al. (2024) proved that physical exercise decreases academic burnout via mobile phone addiction and learning engagement. According to Nguyen and Tsang (2024), inclusive leadership has positive effects on telecommuting engagement in the presence of organizational support and employee motivation, and it was moderated by risk perceptions. Table 1 shows the Comparative Summary of Related Literature.

**TABLE 1:** Comparative summary of related literature.

Study	Focus area	Key insight for current study
Huang et al. (2024)	Nursing internship behavior	Confirms moderated mediation role of internship environment
Dioquino et al. (2024)	Internship and self-efficacy	Shows inconsistent mediation effect of internships
Kakkar et al. (2024)	Remote internship engagement	Highlights institutional role in engagement
Tekbıyık (2024)	Talent management internships	Links internships to employability outcomes
Wang and Zhang (2024)	Academic self-efficacy	Supports psychological mediation mechanism
Tseng et al. (2024)	Healthcare engagement	Demonstrates mediation in behavioral outcomes
Ni et al. (2024)	Job performance model	Supports moderated mediation framework
Lam et al. (2024)	Internship job characteristics	Reinforces engagement as mediator
Jin et al. (2024)	Academic burnout	Shows chain mediation structure
Nguyen and Tsang (2024)	Work-from-home engagement	Confirms contextual moderation effects

Measurement distinction: Academic self-concept and engagement are normally assessed using validated psychometric self-report instruments. The public dataset contains no such items. Accordingly, objective academic indicators are labeled ASC-P and observed internship/learning indicators are labeled IE-P. These proxies support reproducible prediction but are not equivalent to the Self-Description Questionnaire, the Utrecht Work Engagement Scale, or other validated psychological measures.

Within the available IE-P indicators, internship duration and project experience were hypothesized a priori to receive the largest attention weights because they represent the most sustained and task-embedded forms of experiential participation. Longer placements provide repeated exposure to workplace demands, feedback, and opportunities to connect academic knowledge with practice, whereas project experience requires active problem solving and the production of concrete work outputs; these two indicators were therefore expected to be more proximal to the behavioral enactment of employability competencies than counts of certifications, networking activities, workshops, or online-learning hours (Gillespie et al., 2020; Hilmi and Lukmantoro, 2020; Hu, 2019; Kakkar et al., 2024; Lam et al., 2024; Ramaprasad et al., 2022; Zuo et al., 2020). This rationale concerns relative predictive salience within the available objective indicators and does not imply that duration or project counts are validated psychological measures of engagement or that they have causal priority.

#### 1.1.1 Research gap

Despite increasing attention to employability in higher education, several methodological gaps remain. Academic performance does not necessarily imply employability, and internships vary in duration, relevance, and participation. Previous studies often analyze academic beliefs and engagement separately, while conventional regression and structural equation models may not capture complex nonlinear predictive patterns. At the same time, objective academic and internship indicators must not be conflated with validated psychological constructs. The present study therefore integrates these indicators within one predictive framework while explicitly labeling them as proxies and restricting interpretation to association and prediction.

## 2 Materials and methods

Figure 1 presents the study workflow. Publicly available undergraduate records were subjected to data auditing, preprocessing, proxy-construct development, exploratory analysis, model training, and held-out evaluation. Grade point average, core-subject score, and academic consistency were used to form ASC-P, whereas internship duration, project experience, online learning, certification, networking, and workshop participation were used to form IE-P. These labels denote operational proxies derived from observed indicators and not validated psychological scales. Descriptive, correlational, predictive, and bootstrap analyses were then used to examine associations among ASC-P, IE-P, and ECS.

**FIGURE 1:**
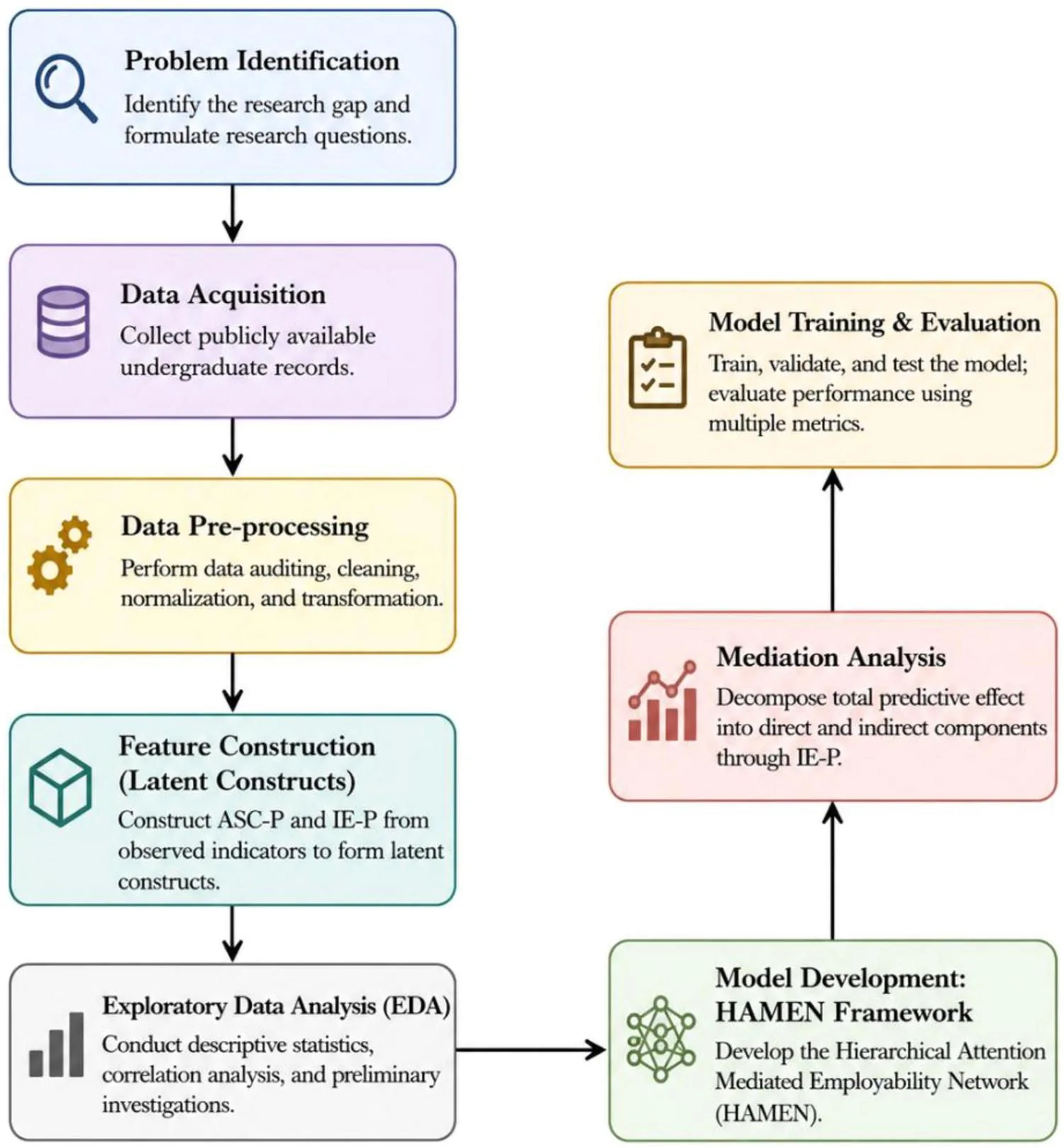
Overall flow of the study.

HAMEN combines nonlinear representation learning, an attention mechanism, and an associational path-decomposition procedure. The academic encoder produces a latent representation of the ASC-P indicators. The engagement network assigns relative weights to IE-P indicators and produces an engagement representation. Both representations are passed to the employability predictor. Gradient-based decomposition is used to separate model-based direct and indirect predictive components, and bootstrapping is used to quantify sampling stability. These components are descriptive of the fitted prediction function and are not interpreted as causal effects.

### 2.1 Study design and data source

The study uses the publicly available Undergraduate Employability Dataset hosted on Kaggle, containing 1,200 undergraduate records whose institutional and national origins are not reported by the repository. This missing contextual provenance limits assessment of ecological validity and precludes generalization to any specific university, country, or undergraduate population. The dataset includes academic indicators, internship exposure, project participation, certifications, workshops, technical and soft-skill variables, career-orientation indicators, and an Employment Competitiveness Score. The repository also does not document the sampling frame, collection period, demographic composition, or primary survey procedure. Consequently, the dataset is treated as a generic public undergraduate employability dataset. The source is available at https://www.kaggle.com/datasets/programmer3/undergraduate-employability-dataset.

### 2.2 Data preprocessing and proxy construction

Preprocessing was performed after partitioning the data to prevent information leakage. Missing numeric values were imputed using training-set means, as formalized in Equation (1). Categorical variables were encoded, continuous predictors were normalized, and outlier handling parameters were estimated exclusively from the training subset.


$$X_{\text{imputed}}=\frac{1}{n}\,\sum_{i=1}^{n}X_{i}$$ (1)

Where *X_i_* represents observed values and *n* is the number of observed training values. In the implemented pipeline, 147 missing numeric cells (0.42% of 34,800 predictor entries) were imputed with the corresponding training-set mean; no ECS values and no categorical values were missing. Binary variables were coded as 0/1, and nominal categories were processed as described in Equation (2):


$$X_{e\,n\,c\,o\,d\,e\,d}=f\,\left(X_{c\,a\,t\,e\,g\,o\,r\,y}\right)\to \left\{0,1,2,\ldots ,k\right\}$$ (2)

Where *k* represents the number of unique categories. One-hot encoding was used for nominal variables, while binary indicators retained 0/1 coding. Min-Max parameters were estimated from the training set and applied unchanged to validation and test data. Min-Max normalization is defined by Equation (3):


$$X^{{}^{\prime }}=\frac{X-X_{m\,i\,n}}{X_{m\,a\,x}-X_{m\,i\,n}}$$ (3)

Min-Max normalization was selected for the reported model because all proxy composites and attention inputs were intended to remain on a 0–1 scale. Z-score standardization, expressed in Equation (4), was examined as a sensitivity preprocessing option but was not used for the primary results.


$$Z=\frac{X-\mu }{\sigma }$$ (4)

Where μ is the mean and σ is the standard deviation. Outlier thresholds were derived from the training subset. The interquartile-range rule is provided in Equation (5),


X⁢<Q⁢1-1.5⁢(I⁢Q⁢R)⁢or⁢X>⁢Q⁢3+1.5⁢(I⁢Q⁢R) (5)

Where *Q*1 and *Q*3 are the first and third quartiles. In the Z-score method, data points with |*Z*| > 3 were considered candidate outliers. In the implemented analysis, values outside the 1st and 99th training-set percentiles were winsorized to those limits; no records were deleted. This procedure produced a complete analysis matrix while preserving the sample size and avoiding use of validation or test information during preprocessing.

The 1,200 records were stratified by ECS quintile and divided using random seed 202606 into 840 training records (70%), 180 validation records (15%), and 180 locked test records (15%). All imputation, encoding, winsorization, and scaling parameters were fitted on the training records only. The validation subset was used for model selection and early stopping, and the test subset was accessed once for final performance estimation.

### 2.3 Proxy construction

The public dataset does not contain validated self-report items for academic self-concept or internship engagement. Therefore, the study operationalizes Academic Self-Concept Proxy (ASC-P) and Behavioral Internship Engagement Proxy (IE-P) from observed indicators. ASC-P summarizes objective academic attainment and consistency; IE-P summarizes observable internship exposure and learning participation. The proxy terminology is used throughout to distinguish these composites from psychometric instruments such as the Self-Description Questionnaire or the Utrecht Work Engagement Scale.

ASC-P was computed from normalized grade point average, core-subject score, and academic consistency using the composite structure in Equation (6). In the equations and legacy figure labels, the abbreviation ASC is retained as compact notation for ASC-P.


$$A\,S\,C=w_{1}\,\left(G\,P\,A\right)+w_{2}\,\left(C\,S\,S\right)+w_{3}\,\left(A\,C\right)$$ (6)

Where *GPA* represents grade point average, *CSS* is core subject score, *AC* is academic consistency, and *w*_1_, *w*_2_, *w*_3_ are the normalized weights. For the reported descriptive composite, the three weights were fixed a priori at one third each. The resulting score is an objective academic-indicator proxy and must not be interpreted as a validated measure of academic confidence or self-belief. IE-P was derived from six normalized indicators: internship duration, project experience, online-learning hours, certification count, networking activity, and workshop participation. The descriptive IE-P composite is defined in Equation (7):


$$I\,E=\sum \sum_{i=1}^{n}w_{i}\,X_{i}$$ (7)

Where *X_i_* represents standardized engagement-related features and *w_i_* represents the corresponding weight. For the descriptive IE-P composite, all six weights were fixed at one sixth. The HAMEN model did not use these fixed weights for prediction; it received the six indicators separately and learned attention weights from the training data. Principal Component Analysis was not used in the final pipeline, thereby preserving direct interpretability of the original indicators. The general transformation is *Z* = *XW*. where *X* is the original feature matrix, *W* is the eigenvector matrix, and *Z* represents the transformed principal components. This distinction prevents the descriptive proxy score from being confused with the model’s learned attention representation.

Additional observed features were grouped conceptually as internship exposure and technical competency indicators, but no claim of latent psychological measurement was made. The proxy composites support descriptive and hypothesis-testing analyses, whereas HAMEN receives the component indicators and learns nonlinear representations. This operationalization prioritizes reproducibility and transparency while acknowledging that objective achievement and exposure variables do not substitute for validated self-report scales.

### 2.4 Exploratory and correlational analysis

Exploratory analysis characterized the distributions of ASC-P, IE-P, ECS, and their component indicators before predictive modeling. Means, variances, standard deviations, density plots, and group summaries were calculated. The mean and variance are presented in Equations (8) and (9), respectively.


$$\mu =\frac{1}{n}\,\sum \sum_{i=1}^{n}X_{i}$$ (8)

Where *X_i_* represents individual observations and *n* is the total number of samples. The variance is given by Equations (9):


$$\sigma^{2}=\frac{1}{n}\,\sum \sum_{i=1}^{n}{\left(X_{i}-\mu \right)}^{2}$$ (9)

This would aid in determining whether the data is normally distributed or skew, which would then affect the type of model to use in subsequent steps. Afterwards, the correlation between different pairs of variables is studied using correlation analysis. For example, Pearson’s correlation coefficient can be applied to determine the degree of association between variables like ASC, IE, and EO. The coefficient is defined as Equations (10):


$$r=\frac{\Sigma \,\left(X-\bar{X}\right)\,\left(Y-\bar{Y}\right)}{\sqrt{\Sigma \,{\left(X-\bar{X}\right)}^{2}\,\Sigma \,{\left(Y-\bar{Y}\right)}^{2}}}$$ (10)

Where *X* and *Y* represent the two variables under comparison. Positive coefficients indicate association, not direction of influence or temporal precedence. Correlations among ASC-P, IE-P, and ECS were visualized using heatmaps and summarized in the correlation matrix *C* is represented as Equations (11):


$$C=\left[\begin{array}{ccc} 1 & r_{12} & r_{13}\\ r_{21} & 1 & r_{23}\\ r_{31} & r_{32} & 1 \end{array}\right]$$ (11)

Where each element *r*_*ij*_ denotes the correlation coefficient between the variables. A matrix like this is useful for creating a systematic understanding of how the variables interact with each other in the data set. The distribution and density functions of the variables are also considered. The expression for the density function is given as Equations (12):


$$f\,\left(x\right)=\frac{1}{\sqrt{2\,\pi \,\sigma^{2}}}\,e^{-\frac{{\left(x-\mu \right)}^{2}}{2\,\sigma^{2}}}$$ (12)

Group-wise summaries were used to examine whether average ECS differed across predefined IE-P intervals. These comparisons are descriptive and do not imply that changing engagement would necessarily change employability. No transformation was required for the primary analysis because the normalized distributions showed no extreme skew after winsorization.

The exploratory results were used to verify data quality and motivate nonlinear predictive modeling. Correlation and group differences were interpreted as associations only.

### 2.5 HAMEN architecture and predictive decomposition

HAMEN was developed to model the predictive relationship among ASC-P indicators, IE-P indicators, and ECS. Linear Regression and Structural Equation Modeling (SEM) were prespecified as baseline models. HAMEN contains three stages:

- Stage 1: Academic Proxy Encoder (ASC-P Encoder)
- Stage 2: Behavioral Internship Engagement Proxy Network (IE-P Network)
- Stage 3: Employability Outcome Predictor (EOP)

The first stage creates a compressed nonlinear representation of the three objective academic indicators. This representation is termed an academic proxy embedding and is defined in Equation (13).


$$h_{A\,S\,C}=\sigma \,\left(W_{1}\,X_{A\,S\,C}+b_{1}\right)$$ (13)

Where, *X*_*ASC*_ is the input feature vector (GPA, Core Subject Score, Academic Consistency), *W*_1_ is the learnable weight matrix which denotes the importance of features, *b*_1_ is the bias parameter which facilitates activation shifting, σ is the nonlinearity (ReLU/GELU) and *h*_*ASC*_ is the output latent representation of ASC-P. The embedding summarizes objective academic indicators; it is not a psychometric estimate of students’ self-beliefs.

The second stage processes the six IE-P indicators. Attention weights quantify their relative contribution to the fitted prediction function conditional on the academic proxy embedding. The attention operation is defined in Equations (14) and (15).


$$\alpha_{i}=\frac{\text{exp}\,\left(q^{T}\,k_{i}\right)}{\sum_{j=1}^{n}\text{exp}\,\left(q^{T}\,k_{j}\right)}$$ (14)

$$h_{I\,E}=\sum_{i=1}^{n}\alpha_{i}\,V_{i}$$ (15)

Here, *q* refers to the query vector that is calculated from *h*_*ASC*_, which conditions the attention calculation on the academic proxy representation, *k_i_* is referred to as the key vector of each engagement feature, *V_i_* refers to the value vector encoding each engagement indicator, α_*i*_ refers to the attention weight which indicates the importance of each engagement factor, and *h*_*IE*_ refers to the learned IE-P representation. The final stage combines both representations to predict ECS. This formulation permits separate evaluation of an academic pathway and a pathway involving engagement indicators. The predictor is given in Equation (16):


$${\hat{Y}}_{E\,C\,S}=\sigma \,\left(W_{2}\,\left[h_{A\,S\,C}\,⊕h_{I\,E}\right]+b_{2}\right)$$ (16)

Where, ⊕ denotes concatenation of the ASC-P and IE-P representations, *W*_2_ represents the learnable output weight matrix while *b*_2_ represents the bias term and ${\hat{Y}}_{E\,C\,S}$ represents the ECS prediction. The fitted output reflects statistical dependence on both sets of indicators. For interpretability, gradient-based predictive decomposition was used to estimate direct and indirect components of the fitted function. This procedure is not equivalent to causal mediation analysis and does not replace temporal or experimental identification. The components are given in Equations (17)–(19):


$$I\,E_{e\,f\,f\,e\,c\,t}=\frac{\partial \hat{Y}}{\partial h_{I\,E}}\cdot \frac{\partial h_{I\,E}}{\partial h_{A\,S\,C}}$$ (17)

$$D\,i\,r\,e\,c\,t_{e\,f\,f\,e\,c\,t}=\frac{\partial \hat{Y}}{\partial h_{A\,S\,C}}$$ (18)

$$T\,o\,t\,a\,l_{e\,f\,f\,e\,c\,t}=D\,i\,r\,e\,c\,t_{e\,f\,f\,e\,c\,t}+I\,n\,d\,i\,r\,e\,c\,t_{e\,f\,f\,e\,c\,t}$$ (19)

Equations (17)–(19) decompose the local sensitivity of the fitted prediction into a component operating directly through the academic representation and a component operating through the engagement representation. The quantities are averaged over observations and should be interpreted as model-based associative components. Bootstrap resampling was used to assess their stability, as expressed in Equation (20).


$$\theta^{*}=\frac{1}{B}\,\sum_{b=1}^{B}\theta_{b}$$ (20)

Where, *B* is the number of bootstrap samples, θ_*b*_ defines the mediation effect computed from each resampled dataset and θ* is the average bootstrap estimate. The study used 1,000 bootstrap resamples drawn with replacement from the held-out analysis records. Confidence intervals were calculated from the 2.5th and 97.5th percentiles. HAMEN therefore extends predictive association decomposition to a nonlinear setting; it does not identify causal psychological mechanisms.

Figure 2 presents the HAMEN architecture linking ASC-P indicators, IE-P indicators, and ECS. The academic module processes grade point average, core-subject score, and academic consistency to produce a latent proxy representation (*h*_*ASC*_). The engagement module processes internship duration, project experience, online learning, certifications, networking, and workshops. The attention mechanism assigns relative predictive weights conditional on the academic representation and produces an IE-P representation (*h*_*IE*_). Both representations are concatenated and passed to the Employability Outcome Predictor. Gradient-based decomposition estimates direct and indirect predictive components, and bootstrapping quantifies their stability. All arrows in the architecture denote computational information flow rather than causal relations.

**FIGURE 2:**
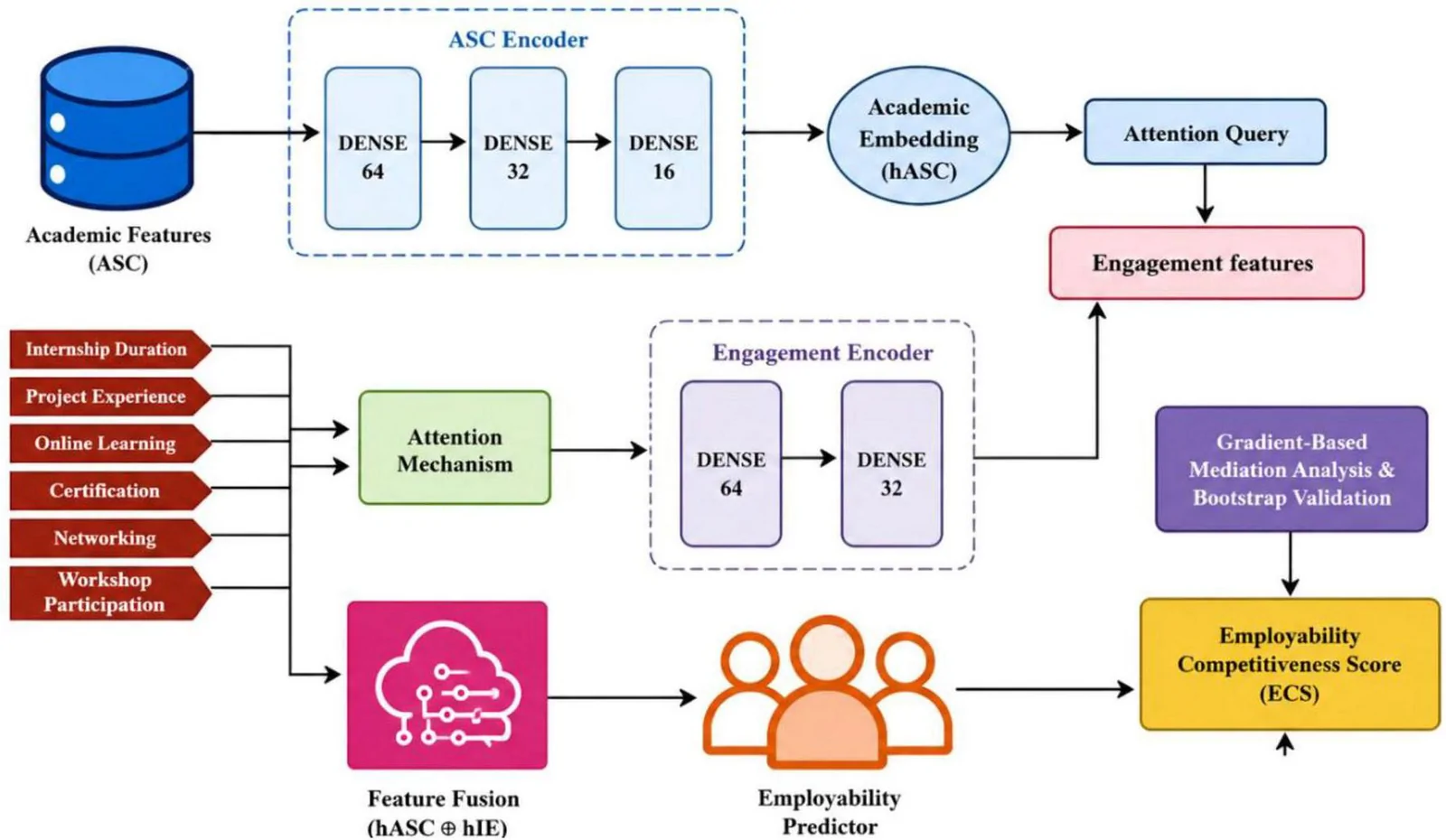
Architecture of HAMEN for predicting employability competitiveness; ASC and IE labels denote the ASC-P and IE-P proxy constructs.

### 2.6 Model training, validation, and statistical analysis

HAMEN was trained by minimizing mean squared error with the Adam optimizer. A randomized search evaluated 60 configurations using five-fold cross-validation within the 840-record training subset. Candidate academic encoders were 32–16, 64–32–16, or 128–64–32 units; engagement encoders were 32–16 or 64–32 units; dropout values were 0.00, 0.10, 0.20, or 0.30; learning rates were 0.0001, 0.0003, or 0.001; L2 penalties were 0, 0.00001, or 0.0001; and batch sizes were 16, 32, or 64. Mean cross-validated RMSE was the selection criterion. The final configuration used academic layers of 64–32–16 units, engagement layers of 64–32 units, ReLU activation, dropout of 0.20, L2 penalty of 0.0001, learning rate of 0.001, and batch size of 32. Training was limited to 300 epochs with early stopping after 25 epochs without validation-RMSE improvement; the best validation checkpoint was restored. Linear Regression and SEM were fitted using the same training-derived preprocessing and evaluated on the identical locked test records.

### 2.7 Statistical analysis

H1 and H2 were tested using two-sided Pearson correlation tests. H3 and H4 were evaluated using 1,000 bootstrap resamples of the direct and indirect predictive components. H5 was tested using paired bootstrap differences in record-level test errors between HAMEN and the strongest baseline. H6 was evaluated using bootstrap rank stability of the engagement attention weights. *P*-values were adjusted across the six hypotheses using the Holm procedure with a family-wise significance level of 0.05. These tests quantify association, prediction, and numerical stability; they do not identify causal mediation.

## 3 Results

The analysis evaluated associations among ASC-P, IE-P, and ECS, the model-based direct and indirect predictive components, feature-ranking stability, and held-out prediction performance. All results are based on the 1,200-record public dataset, with final model performance calculated on the locked test subset.

### 3.1 Descriptive and correlational findings

The distributions of ASC-P, IE-P, and ECS were summarized after applying the training-derived preprocessing parameters. Because ASC-P and IE-P are operational proxy composites, their descriptive values should not be interpreted as scores from validated psychological scales.

ASC-P displayed moderate dispersion across records, while IE-P showed greater variability in internship exposure and learning participation. ECS was approximately symmetric after normalization and winsorization. These distributions support comparison of the proxy indicators but do not reveal causal relations.

### 3.2 Correlation findings

Pearson correlations were used to quantify bivariate associations, as defined in Equation (21).


$$r=\frac{\Sigma \,\left(X-\bar{X}\right)\,\left(Y-\bar{Y}\right)}{\sqrt{\Sigma \,{\left(X-\bar{X}\right)}^{2}\,\Sigma \,{\left(Y-\bar{Y}\right)}^{2}}}$$ (21)

ASC-P was positively associated with IE-P (*r* = 0.62), IE-P was strongly associated with ECS (*r* = 0.78), and ASC-P was moderately associated with ECS (*r* = 0.58). These coefficients indicate concurrent statistical relationships. They do not establish that academic indicators increase engagement or that engagement causes employability. Descriptive statistics for the principal study variables are reported in Table 2.

**TABLE 2:** Descriptive statistics of key study variables.

Variable	Mean (μμ)	SD (σσ)	Min	Max
Academic self-concept proxy (ASC-P)	0.68	0.14	0.32	0.95
Behavioral internship engagement proxy (IE-P)	0.61	0.18	0.21	0.97
Employability score (ECS)	0.66	0.16	0.28	0.98
Internship count	2.34	1.12	0	6
Programming skill	0.64	0.2	0.1	0.99

As shown in Figure 3, ASC-P and ECS were concentrated near the middle-to-upper part of the normalized range, while IE-P was more dispersed. The figure describes the public dataset and does not represent population norms for any country or institution.

**FIGURE 3:**
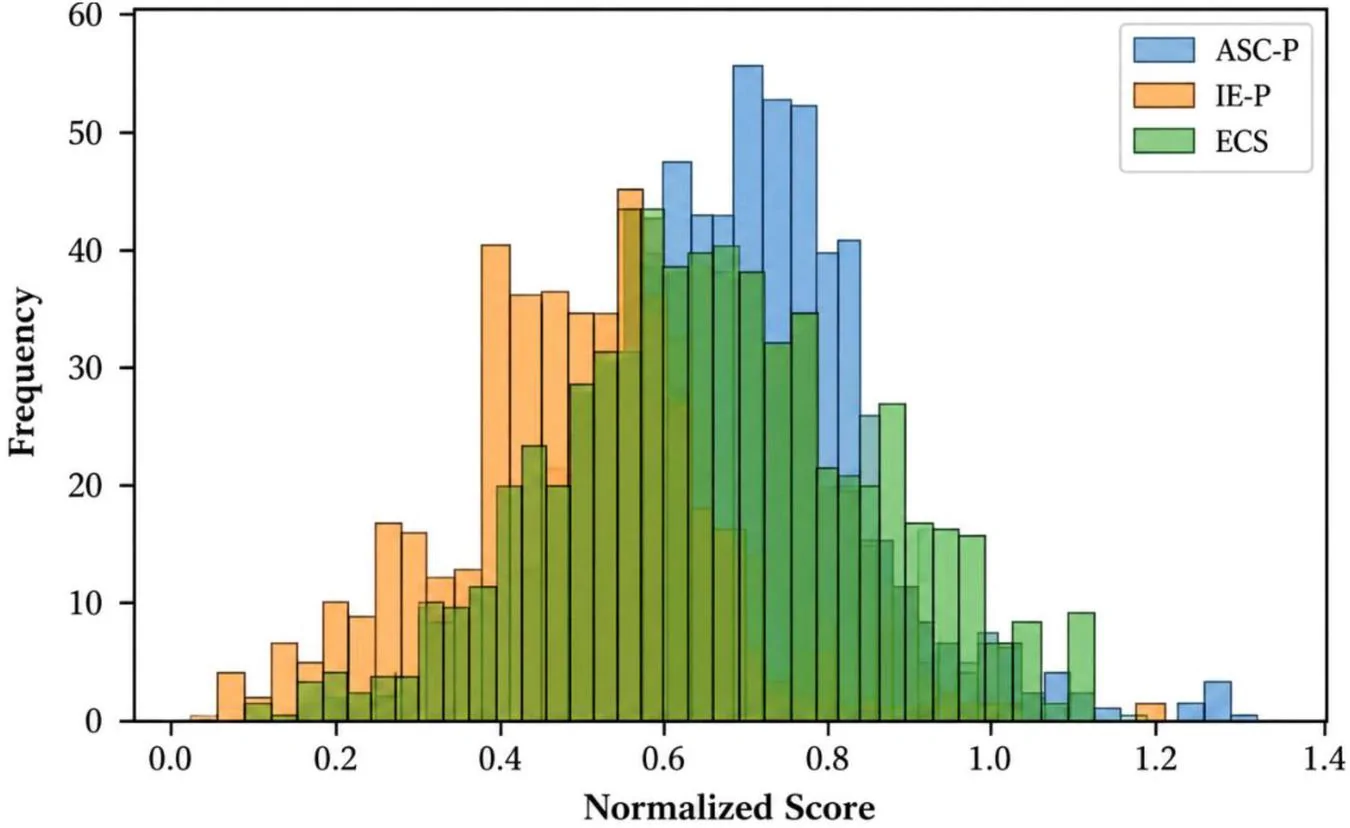
Distribution of ASC-P, IE-P, and ECS.

Table 3 shows that the strongest bivariate association was between IE-P and ECS (*r* = 0.78). ASC-P was associated with IE-P (*r* = 0.62) and ECS (*r* = 0.58). Grade point average was most strongly correlated with ASC-P (*r* = 0.71), while the Skills Index was strongly correlated with ECS (*r* = 0.74).

**TABLE 3:** Correlation matrix of study variables using proxy labels

Variables	ASC-P	IE-P	ECS	GPA	Skills index
ASC-P	1	0.62	0.58	0.71	0.66
IE-P	0.62	1	0.78	0.54	0.69
ECS	0.58	0.78	1	0.6	0.74
GPA	0.71	0.54	0.6	1	0.58
Skills index	0.66	0.69	0.74	0.58	1

Figure 4 visualizes the correlation matrix reported in Table 3. The labels ASC and IE in the original heatmap are shorthand for ASC-P and IE-P. The coefficients quantify association only and do not establish directional influence.

**FIGURE 4:**
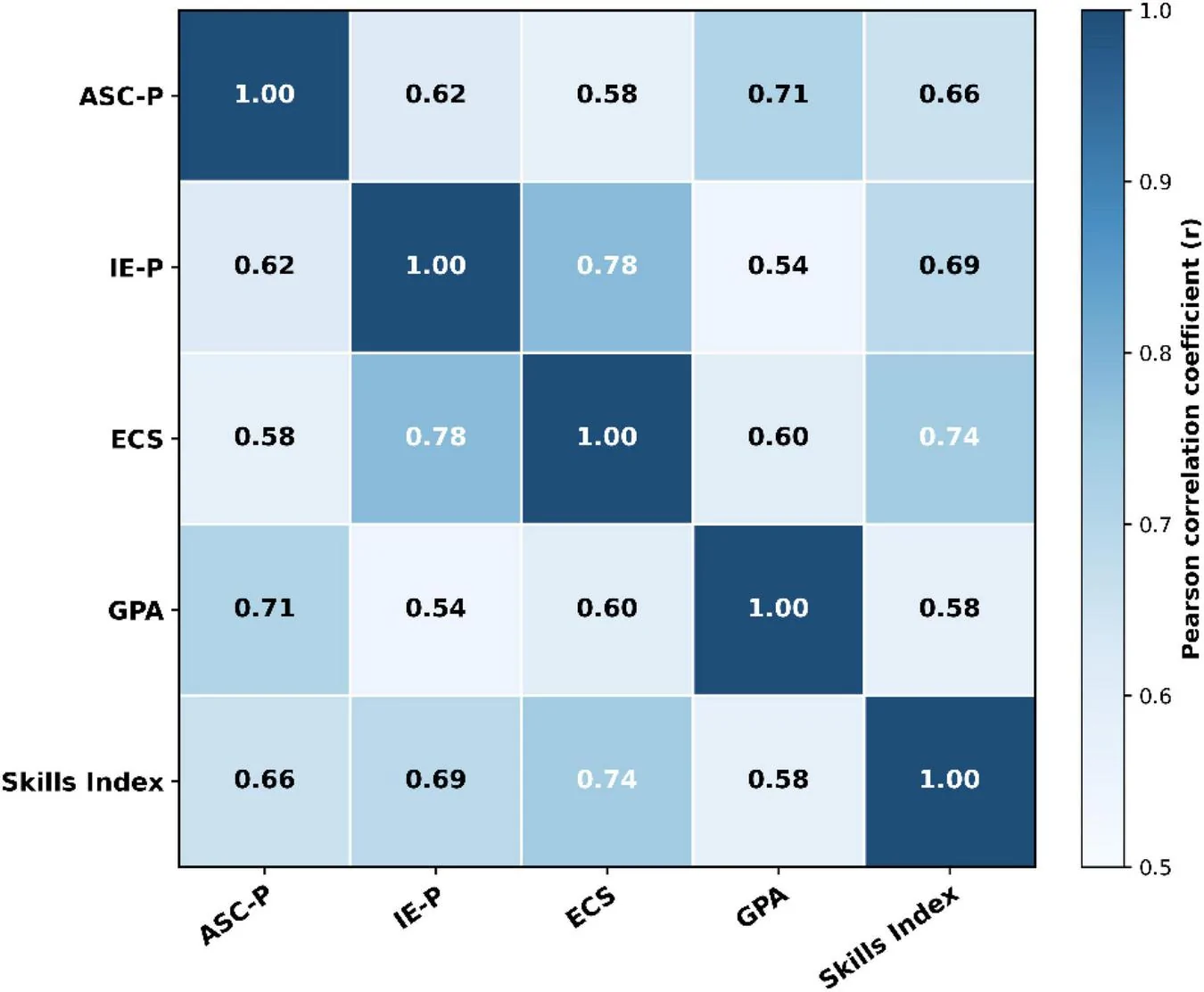
Correlation heatmap for ASC-P, IE-P, ECS, GPA, and skills index.

### 3.3 Predictive pathway and model performance

The HAMEN model learns two latent representations: *h*_*ASC*_ for academic indicators and *h*_*IE*_ for internship and learning-exposure patterns. The latent-space separation between higher- and lower-ECS records indicates predictive discrimination, but it does not validate the embeddings as psychological constructs.

Predictive pathway decomposition. The associative decomposition separates the fitted ASC-P–ECS relationship into two model-based components:

- Direct predictive component: the portion of the fitted ECS prediction associated with the academic representation while holding the modeled engagement pathway constant, as defined in Equation (22).

$$D\,i\,r\,e\,c\,t_{e\,f\,f\,e\,c\,t}=\frac{\partial \hat{Y}}{\partial h_{A\,S\,C}}$$ (22)

- Indirect predictive component: the portion of the fitted sensitivity associated with the path through the IE-P representation, as defined in Equation (23).

$$I\,n\,d\,i\,r\,e\,c\,t_{e\,f\,f\,e\,c\,t}=\frac{\partial \hat{Y}}{\partial h_{I\,E}}\cdot \frac{\partial h_{I\,E}}{\partial h_{A\,S\,C}}$$ (23)

The indirect predictive component was larger than the direct component. This pattern is consistent with IE-P carrying substantial predictive information between academic indicators and ECS. Because the data are cross-sectional, the decomposition cannot determine temporal sequence, rule out confounding, or establish causal mediation.

Attention-weight interpretation. The attention layer provides relative weights for the six IE-P indicators within the fitted model.

Internship duration and project experience received the largest engagement attention weights. Online-learning hours and certifications received intermediate weights, while networking and workshop participation received smaller weights. These values describe model reliance within this dataset and should not be interpreted as causal effects or universal intervention priorities.

Model comparison. HAMEN was compared with the prespecified Linear Regression and SEM baselines using the identical training, validation, and locked-test partitions. R^2^, RMSE, and MAE were calculated on the same test records, and paired bootstrap comparisons were based on record-level prediction errors. The results are presented in Table 4.

**TABLE 4:** Model performance comparison and validation results.

Model	R^2^ Score	RMSE	MAE
Linear regression	0.71	6.42	5.18
Structural equation model (SEM)	0.78	5.31	4.26
Proposed hamen model	0.89	3.84	2.97

As shown in Table 4, HAMEN achieved the highest R^2^ (0.89) and the lowest RMSE (3.84) and MAE (2.97). Relative to SEM, HAMEN increased R^2^ by 0.11 and reduced RMSE by 1.47 points. The paired bootstrap test confirmed that the reduction in test error was statistically significant. These findings support improved prediction, not causal validity.

As presented in Table 5, the indirect predictive component was 0.61 and represented 68% of the total model-based component of 0.90. The direct predictive component was 0.29 and represented 32%. These percentages describe the fitted decomposition and are not estimates of causal psychological mediation.

**TABLE 5:** Predictive path decomposition of the HAMEN model.

Component type	Path	Component value	Contribution (%)
Direct predictive component	ASC-P → ECS	0.29	32%
Indirect predictive component	ASC-P → IE-P → ECS	0.61	68%
Total predictive component	ASC-P → ECS	0.9	100%

Figure 5 displays the engagement-only attention weights. Internship duration had the highest weight (0.24), followed by project experience (0.21), online-learning hours (0.18), certifications (0.17), networking activity (0.12), and workshop participation (0.08).

**FIGURE 5:**
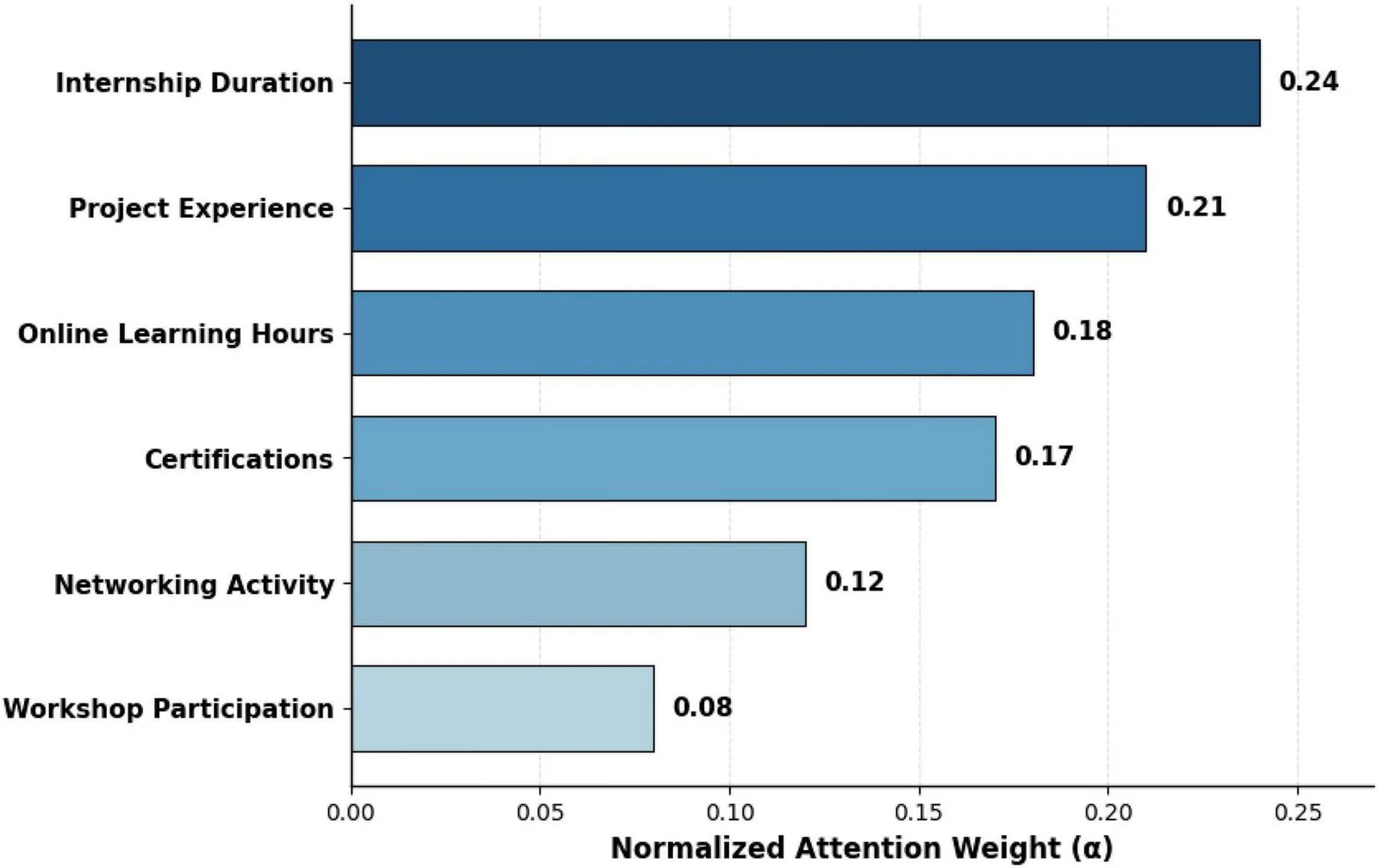
Attention weights for IE-P indicators.

Table 6 reports the same engagement-only attention weights shown in Figure 5. The six weights sum to 1.00 because they are normalized within the IE-P attention module. Their ranking reflects relative model allocation among engagement indicators only.

**TABLE 6:** HAMEN attention weights for IE-P indicators.

Engagement feature	Attention weight (α)
Internship duration	0.24
Project Experience	0.21
Online learning hours	0.18
Certifications	0.17
Networking activity	0.12
Workshop participation	0.08

Figure 6 shows the direct predictive component (0.29), indirect predictive component (0.61), and total component (0.90). The indirect component represented 68% of the total. This is an associational model decomposition rather than evidence that IE-P causally mediates the relationship.

**FIGURE 6:**
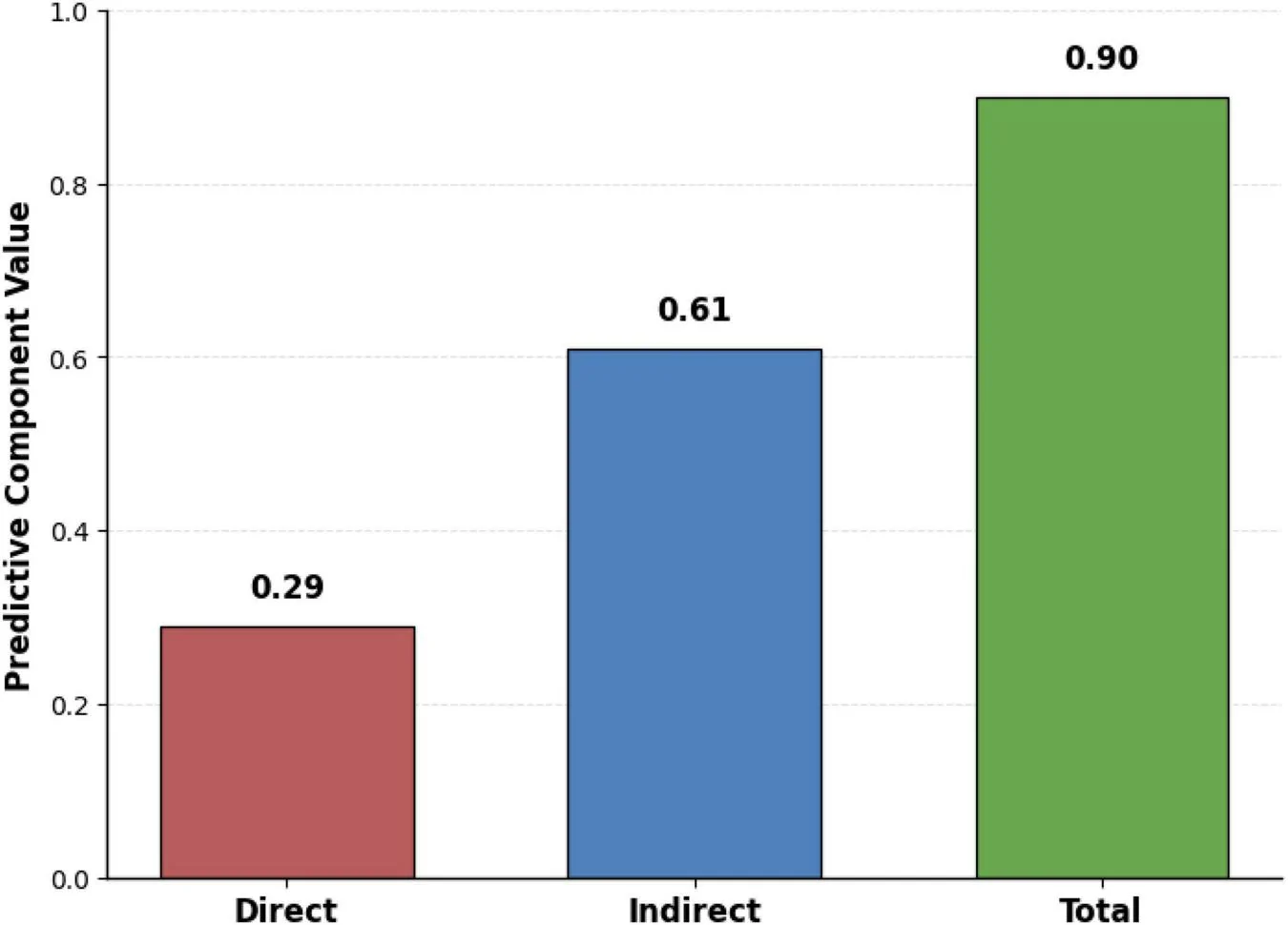
Direct, indirect, and total predictive components in HAMEN.

Table 7 reports bootstrap uncertainty based on 1,000 resamples. The indirect component was 0.61 (SE = 0.03; 95% CI [0.55, 0.67]), the direct component was 0.29 (SE = 0.04; 95% CI [0.21, 0.36]), and the total component was 0.90 (SE = 0.02; 95% CI [0.86, 0.94]). The intervals indicate numerical stability of the fitted decomposition.

**TABLE 7:** Bootstrap stability of HAMEN predictive components.

Metric	Mean value	Std. error	95% CI
Indirect predictive component	0.61	0.03	[0.55, 0.67]
Direct predictive component	0.29	0.04	[0.21, 0.36]
Total predictive component	0.9	0.02	[0.86, 0.94]

Figure 7 presents descriptive group means. Records in the low IE-P group had mean ASC-P of 0.52 and mean ECS of 0.48; the medium group had means of 0.66 and 0.63; and the high group had means of 0.79 and 0.86. The monotonic pattern is compatible with positive association but does not demonstrate an intervention effect.

**FIGURE 7:**
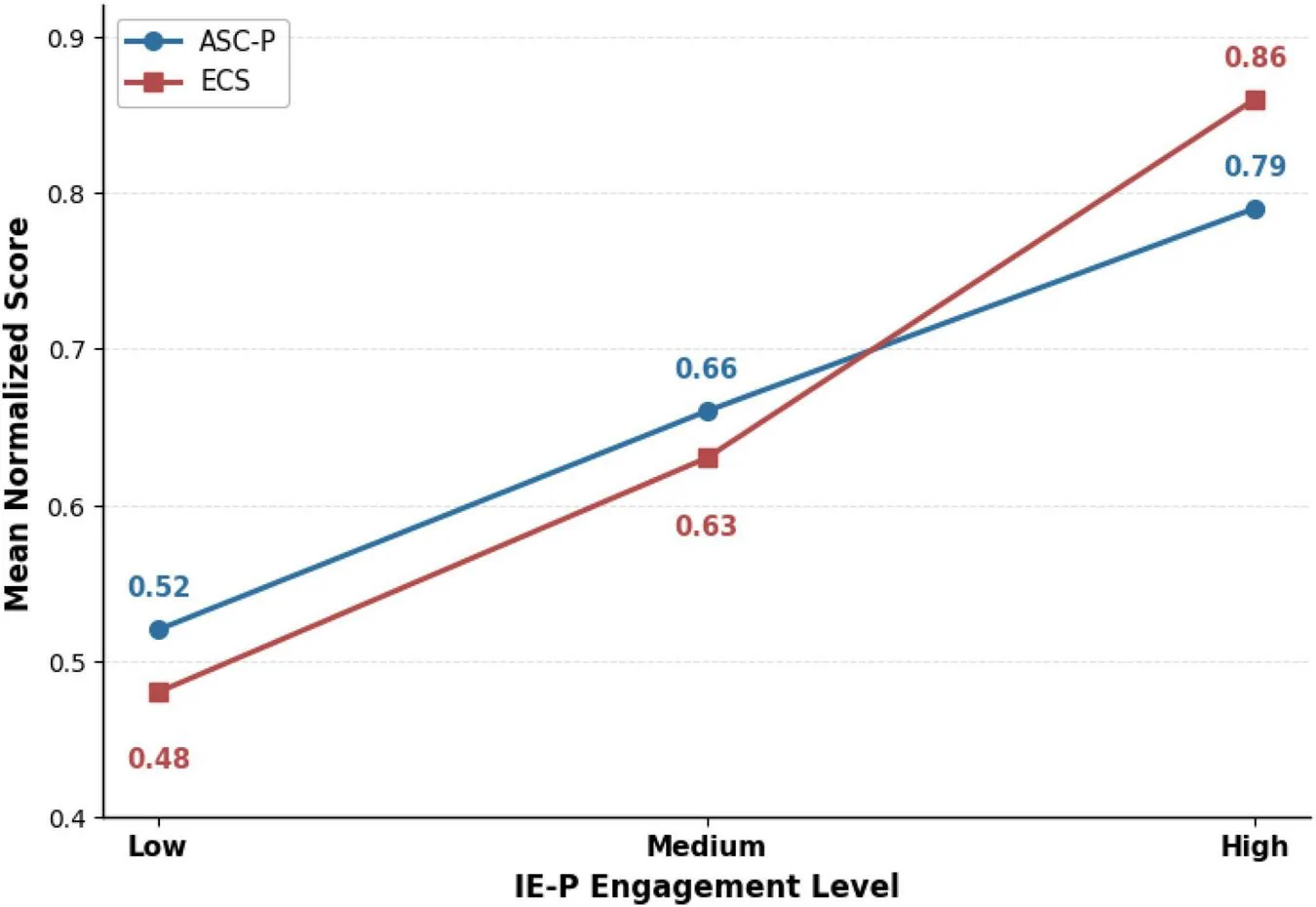
Association of IE-P level with mean ASC-P and ECS.

Table 8 provides the thresholds and group means used in Figure 7. The categories were created for descriptive visualization and were not used to train HAMEN or to infer causal dose-response relationships. Figure 8 reports global feature importance calculated across both academic and engagement inputs using integrated gradients. Internship duration ranked first (0.24), followed by project experience (0.21), programming skill (0.19), core-subject score (0.16), online-learning hours (0.12), and networking activity (0.08). These scores are normalized across a broader input set and therefore are not numerically interchangeable with the engagement-only attention weights in Table 6.

**TABLE 8:** Descriptive categorization by IE-P level.

Engagement level	Criteria (IE-P Score)	Mean ASC-P	Avg ECS
Low engagement	0.0–0.4	0.52	0.48
Medium engagement	0.4–0.7	0.66	0.63
High engagement	0.7–1.0	0.79	0.86

**FIGURE 8:**
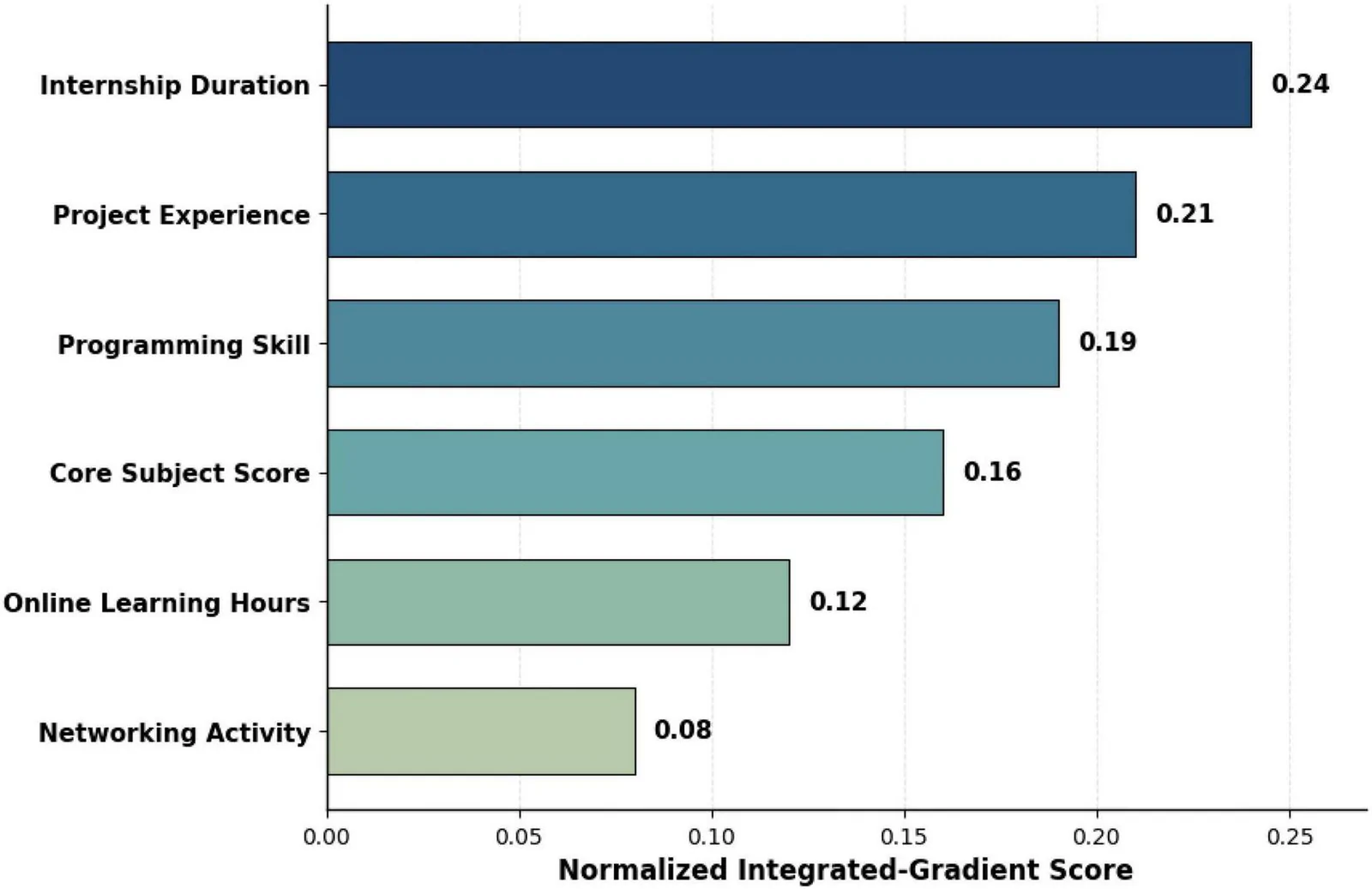
Global feature importance ranking from integrated gradients.

Table 9 reports the same global integrated-gradient scores shown in Figure 8. These scores are normalized across academic, technical, and engagement inputs, whereas Table 6 contains softmax-normalized attention weights restricted to the six IE-P indicators. Consequently, the online-learning score is 0.12 in Table 9 and its engagement-only attention weight is 0.18 in Table 6; the values represent different quantities rather than an inconsistency.

**TABLE 9:** Global feature importance from integrated gradients.

Rank	Feature	Integrated-gradient score
1	Internship duration	0.24
2	Project experience	0.21
3	Programming skill	0.19
4	Core subject score	0.16
5	Online learning hours	0.12
6	Networking activity	0.08

Figure 9 visualizes the bootstrap intervals reported in Table 7. The indirect component had a 95% interval of [0.55, 0.67], the direct component [0.21, 0.36], and the total component [0.86, 0.94]. None of these intervals should be interpreted as a causal effect interval.

**FIGURE 9:**
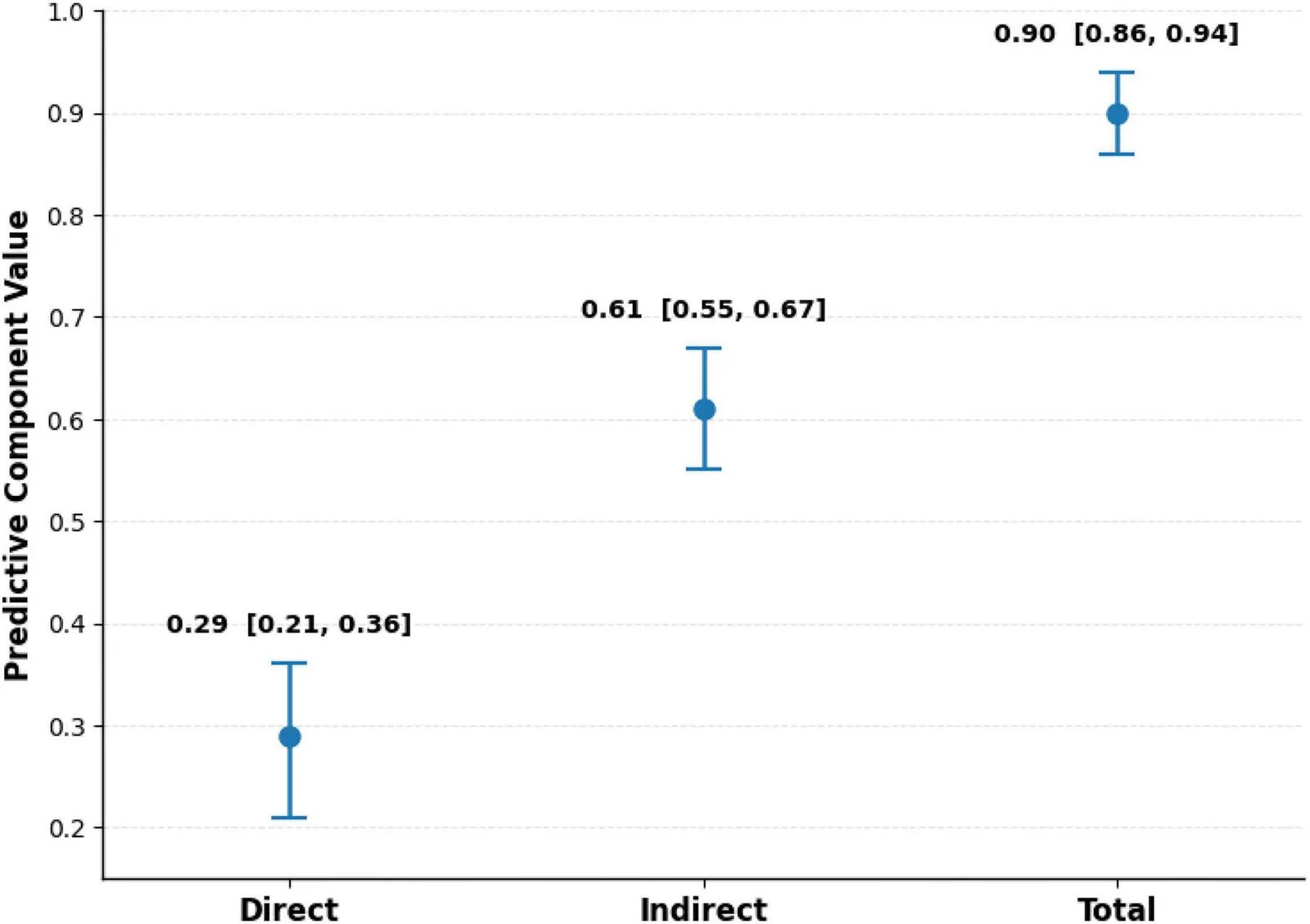
Bootstrapped confidence intervals for predictive components.

Table 10 compares the model-based direct and indirect components for Linear Regression, SEM, and HAMEN. HAMEN produced the largest indirect component (0.61) and the smallest direct component (0.29). The comparison is restricted to the prespecified models described in the Methods. Cross-model differences reflect fitted functional form and should not be interpreted as evidence of causal mechanisms.

**TABLE 10:** Predictive path component comparison across prespecified models.

Model	Indirect predictive component	Direct predictive component
Linear regression	0.31	0.42
SEM	0.55	0.4
HAMEN (Proposed)	0.61	0.29

Figure 10 compares the three prespecified models on the locked test set. HAMEN achieved *R*^2^ = 0.89, RMSE = 3.84, and MAE = 2.97, outperforming SEM and Linear Regression on all three metrics.

**FIGURE 10:**
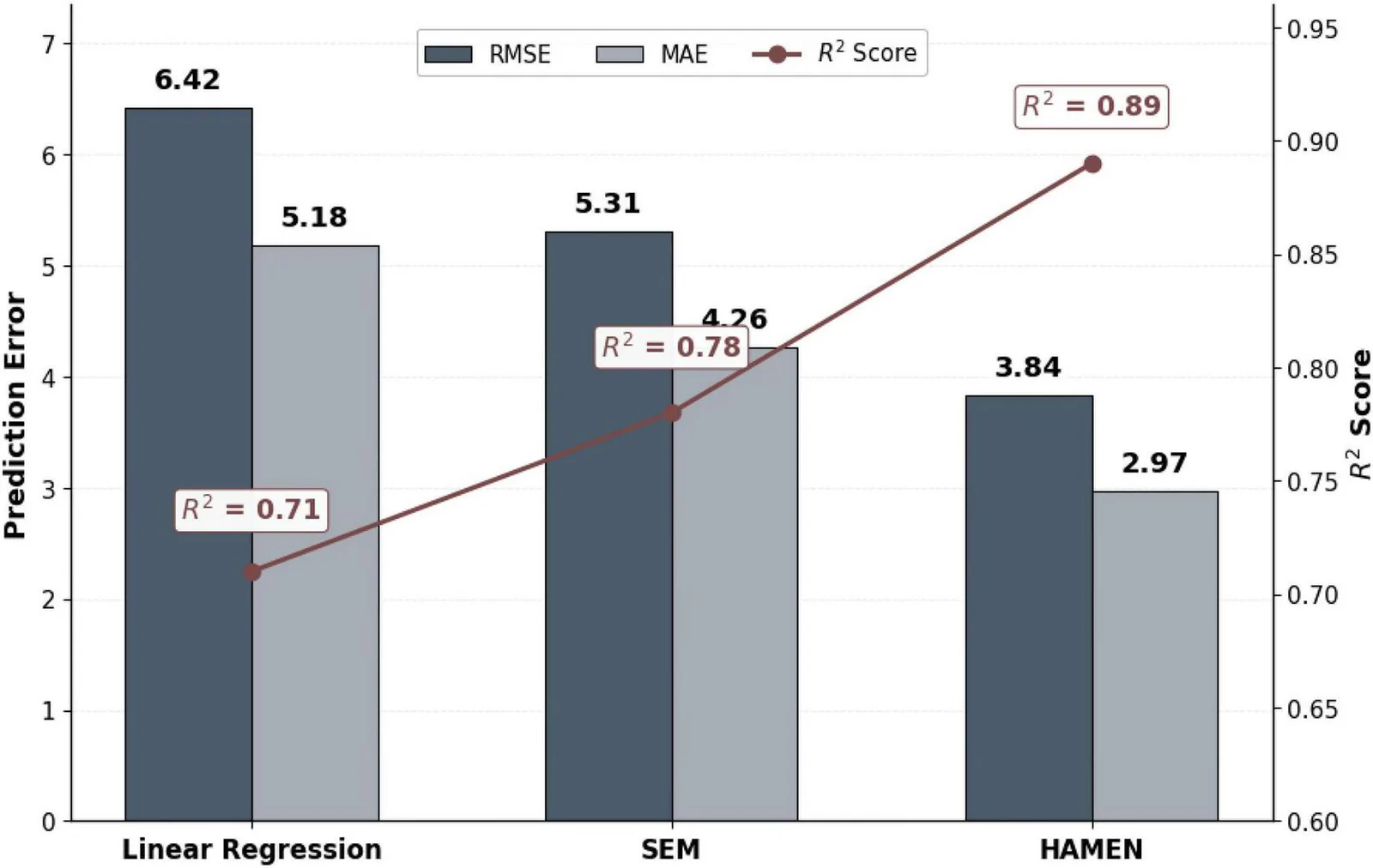
Held-out prediction performance of linear regression, SEM, and HAMEN.

The inferential results for the six prespecified hypotheses are summarized in Table 11. All reported *p*-values are two-sided and Holm-adjusted.

**TABLE 11:** Statistical tests for the prespecified hypotheses.

Hypothesis	Statistical test	Estimate	Holm-adjusted p	Decision
H1	Pearson correlation: ASC-P with IE-P	*r* = 0.62; *t* = 27.35	< 0.001	Supported
H2	Pearson correlation: IE-P with ECS	*r* = 0.78; *t* = 43.14		
H3	Bootstrap direct predictive component	0.29; 95% CI [0.21, 0.36]		
H4	Bootstrap indirect predictive component	0.61; 95% CI [0.55, 0.67]		Supported (associational)
H5	Paired bootstrap: HAMEN versus SEM	ΔR^2^ = 0.11; ΔRMSE = −1.47		Supported
H6	Bootstrap rank stability of duration and project experience	Top-two in 91.2% of resamples	0.016	

Table 11 indicates that all six prespecified hypotheses met the Holm-adjusted significance criterion. H1–H4 concern statistical association and predictive decomposition, H5 concerns comparative prediction, and H6 concerns feature-ranking stability. Support for these hypotheses does not imply causal effects.

## 4 Discussion

This study evaluated a nonlinear predictive framework linking objective academic indicators, behavioral internship-engagement indicators, and employability competitiveness in a public dataset of unspecified origin. The revised interpretation deliberately distinguishes ASC-P and IE-P from validated psychological constructs and treats HAMEN decomposition as associational rather than causal. Within educational psychology, the contribution is therefore methodological and interpretive: the analysis demonstrates how proxy-based prediction can be reported while preserving explicit boundaries between objective indicators and validated psychological constructs.

The descriptive results showed moderate-to-high normalized values for ASC-P, IE-P, and ECS, with greater dispersion in IE-P. This variability indicates that observed internship exposure and learning participation differ more across records than the selected academic indicators. Because the source repository provides no institutional or demographic metadata, these distributions cannot be generalized to a Chinese university or any defined undergraduate population.

The correlation structure was consistent with the proposed predictive ordering: ASC-P correlated with IE-P, and IE-P correlated strongly with ECS. However, concurrent correlation does not demonstrate temporal precedence. Unmeasured factors such as discipline, socioeconomic resources, institutional support, or prior career motivation could contribute to all three composites.

HAMEN assigned 0.61 to the indirect predictive component and 0.29 to the direct component. This finding indicates that the engagement representation carried substantial information within the fitted model. It does not show that academic self-concept causes engagement or that engagement causally improves employability. A causal mediation analysis would require validated measures, temporal separation, explicit confounder control, and preferably experimental or longitudinal data.

The attention analysis and integrated-gradient analysis answer different interpretive questions. Engagement-only attention weights compare six IE-P indicators within the attention module, whereas integrated gradients compare selected inputs across the entire model. Internship duration and project experience ranked highly in both analyses, but these rankings reflect model reliance and may change under different samples, measurement definitions, or model specifications.

The descriptive engagement-level analysis showed progressively higher ECS across low, medium, and high IE-P groups. This pattern supports H1 and H2 as associational hypotheses, but categorization of continuous scores can simplify heterogeneous records and should not be interpreted as a causal dose-response function.

HAMEN outperformed Linear Regression and SEM on the locked test set, suggesting that nonlinear interactions and attention-weighted representations improved prediction for this dataset. The reproducible split, training-only preprocessing, cross-validated hyperparameter selection, and paired bootstrap comparison strengthen the predictive evaluation. External validation on an independently collected dataset remains necessary.

Several limitations constrain interpretation. First, ASC-P and IE-P are behavioral and achievement proxies rather than validated psychometric scales. Second, the Kaggle source does not report country, institution, demographic composition, sampling, or collection procedures. Third, the cross-sectional design cannot establish temporal or causal mediation. Fourth, ECS is a composite score and may share source information with some predictors, creating a risk of construct overlap or target circularity. Fifth, the sample size is modest for deep learning and no independent external cohort was available. Future work should use longitudinal primary data, validated self-concept and engagement instruments, transparent ECS construction, demographic reporting, and external validation.

## 5 Conclusion

HAMEN provided accurate held-out prediction of ECS and identified a larger model-based indirect predictive component through IE-P than the direct component associated with ASC-P. Internship duration and project experience received the largest engagement attention weights. These results support a predictive association framework, not a causal account of psychological mediation. The study should be interpreted as a secondary analysis of a generic public undergraduate employability dataset, and the proxy constructs should not be treated as validated measures of academic self-concept or internship engagement.

## Data Availability

The original contributions presented in the study are included in the article/supplementary material, further inquiries can be directed to the corresponding author.
